# Natural Polyphenols as Targeted Modulators in Colon Cancer: Molecular Mechanisms and Applications

**DOI:** 10.3389/fimmu.2021.635484

**Published:** 2021-02-16

**Authors:** Jing Long, Peng Guan, Xian Hu, Lingyuan Yang, Liuqin He, Qinlu Lin, Feijun Luo, Jianzhong Li, Xingguo He, Zhiliang Du, Tiejun Li

**Affiliations:** ^1^Hunan Provincial Key Laboratory of Animal Intestinal Function and Regulation, Laboratory of Animal Nutrition and Human Health, College of Life Sciences, Hunan Normal University, Changsha, China; ^2^Key Laboratory of Agro-ecological Processes in Subtropical Region, Hunan Provincial Key Laboratory of Animal Nutritional Physiology and Metabolic Process, Institute of Subtropical Agriculture, Chinese Academy of Sciences, Changsha, China; ^3^College of Animal Science and Technology, Hunan Agricultural University, Changsha, China; ^4^National Engineering Laboratory for Deep Process of Rice and Byproducts, Hunan Key Laboratory of Grain-oil Deep Process and Quality Control, College of Food Science and Engineering, Central South University of Forestry and Technology, Changsha, China; ^5^Changsha Green Leaf Biotechnology Co., Ltd., Changsha, China; ^6^Cloud Computing Center, Chinese Academy of Sciences, Dongguan, China

**Keywords:** natural polyphenols, colon cancer, targeted modulator, inflammation, microbe

## Abstract

Colon cancer commonly develops from long-term chronic inflammation in the intestine and seriously threatens human health. Natural polyphenols have been valued as a crucial regulator of nutrient metabolism and metabolic diseases, owing to their anti-inflammatory and antioxidant functions and the ability to maintain a balance between gut microbes and their hosts. Notably, experimental and clinical evidence has shown that natural polyphenols could act as a targeted modulator to play a key role in the prevention or treatment of colon cancer. Thus, in this review, we summarized recent advances in the possible regulatory mechanism and the potential application of natural polyphenols in colon cancer, which might be regarded as a novel platform for the colon cancer management.

## Introduction

Colon cancer is one of the most common malignant tumors, which seriously affects the health of modern people. With the change of people's living habits and diet, the incidence of colon cancer is increasing year by year. There are 1.1 million new cases and about 500,000 deaths worldwide every year (1), and the age of onset is younger (2). Colon cancer originates from chronic inflammatory sites or tumor stem cells. On the one hand, colon cancer may originate in a chronically inflamed area with an imperfect repair mechanism that causes normal cell mutations, metastases, and colonization in the colon, resulting in the development of cancer (3, 4). For example, some of genetic, pharmacological and epidemiological data showed that inflammatory bowel disease (IBD) increase risk of colon cancer (5). The development of colon cancer induced by IBD is thought to be multifaceted, with inflammation, injury, increased epithelial cell proliferation rate (6). Proactive measures should be taken to prevent the progression of IBD to colon cancer. On the other hand, recent studies have revealed that colorectal cancers are mainly originated from cancer stem cells or stem cell-like cells (7). The main pathogenesis of cancer stem cells is derived from the disorder of expression of genetic and epigenetic genes involved in inactivation of tumor suppressor genes and activation of oncogenes.

Traditional therapies, such as surgery, chemotherapy, and radiotherapy have great effects as cancer treatments, drug resistance and toxicities remain as major problems in the treatment of patients with cancer. Therefore, the best available and safest therapies are probably nutritional regulation, involving natural polyphenol products, functional amino acids, and numerous dietary treatments, which can help to improve the symptoms of colon cancer and the quality of life of the patients with colon cancer (8, 9). Ohno et al. demonstrated that curcumin could be considered as an effective therapy for treating IBD by improving the abundance of butyrate-producing bacteria and suppressing NF-κB activation (10). Quercetin administration could upregulate the expression of tight junction proteins (i.e., ZO-1, occludin), preventing damage to indomethin-induced Caco-2 cells (11). Many *in vivo* or *in vitro* experiments have also confirmed that polyphenols and polyphenol-rich whole foods are capable of elevating butyrate producers and probiotics, alleviating colitis (12). Therefore, in this review, we summarize the recent advances in the possible regulatory mechanism and potential application of natural polyphenols, which would be a juncture point of nutrition and medicine that holds considerable promise in treating colon cancer.

## Types and Metabolism of Polyphenols

More than 8,000 natural polyphenol products have been found in the flowers, roots, leaves, and fruits of plants, which are considered as secondary metabolites and primarily exist as the benzene rings that bind to multiple hydroxyl groups or are sometimes linked to aromatic carbon (13). According to the different substituents on benzene rings, natural polyphenol products can be divided into flavonoids (i.e., flavones, flavonols, isoflavones, neoflavonoids, chalcones, and anthocyanidins) and non-flavonoids (i.e., phenolic acids, stilbenoids, and phenolic amides) (Table 1) (19). It has been reported that polyphenols are produced by the shikimate or acetate pathway and possess numerous positive functions, including anti-inflammatory and antioxidant effects, maintaining intestinal microecological balance, and inhibiting cell apoptosis (18, 20–23).

**Table 1:** The main sources of polyphenols in the diet.

**Items**	**Dietary sources**
**Flavonoids** (14–17)	
Flavones	Onion, apple, cherry, broccoli, tomato
Flavonols	Fruits, vegetables, and beverages, beer, tea, cocoa, pulses, spices
Flavanones	Tomatoes, pulses, aromatic plants, aromatic plants
Flavanols	Tea, red wine, chocolate, skins of grape, skins of apple, blueberry
Isoflavonoids	soy, leguminous plants
Anthocyanidins	Flower petals, fruits, vegetables, varieties of grains, black rice
Proanthocyanidins	Berries, tea, wine, pommes, drupes, nuts, pulses
**Non-flavonoids** (16, 18)	
Phenolic acids	Wine, coffee, apple, cocoa, tea, vegetables, fruits spices, aromatic plants
Stilbenoids	Cocoa, red wine, peanuts, grapes, almonds

**Figure 1:**
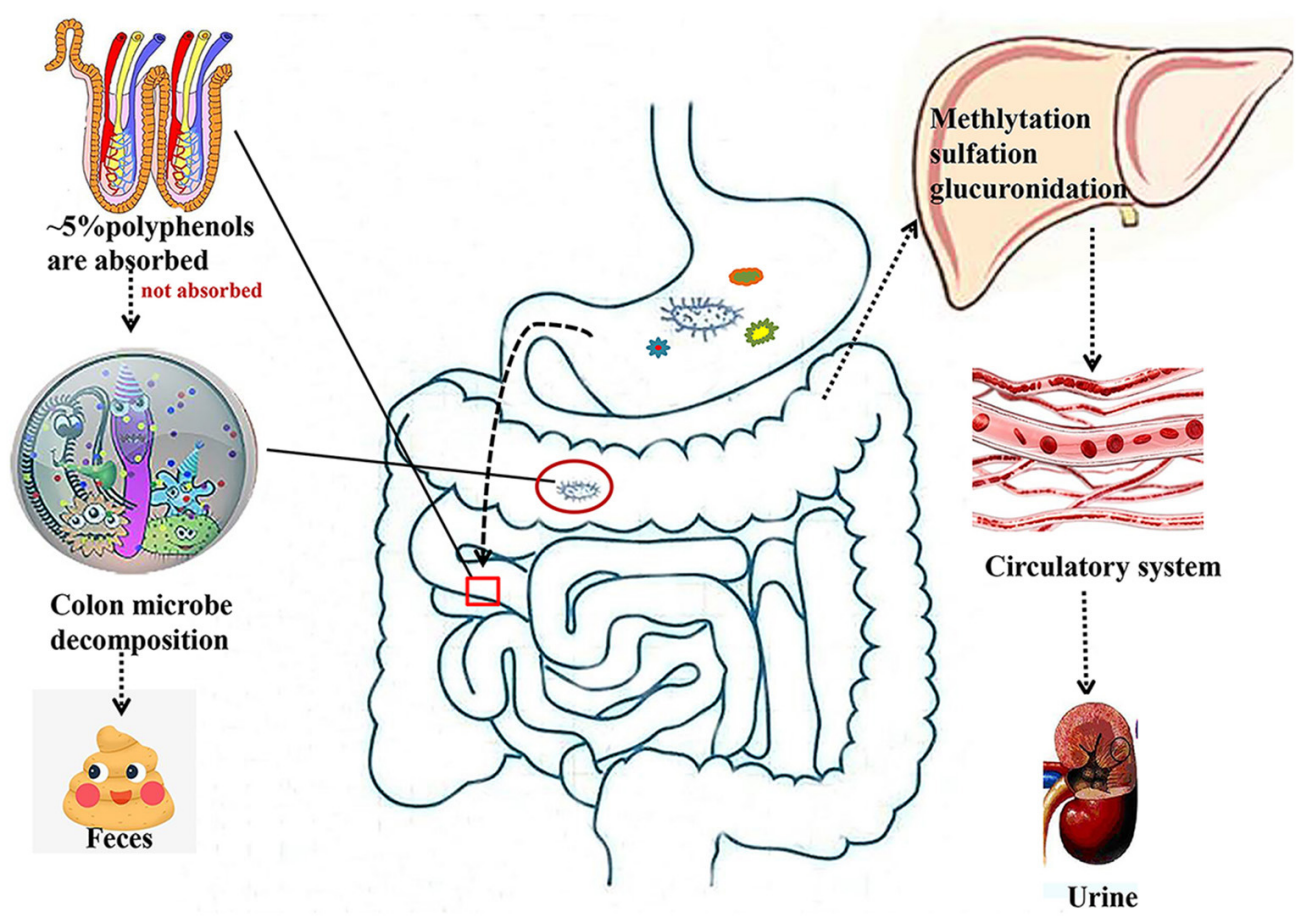
Absorption and metabolism of natural polyphenols. Most polyphenols are metabolized by microorganisms in the colon into small molecules that enter the liver and then circulate through the bloodstream into the circulatory system.

It is known that only 5–10% of polyphenols are absorbed in the intestine, while 90–95% of polyphenols are absorbed in other tissues, such as the liver, adipose, and skeletal muscle tissues (Figure 1). Gut microbes play a key role in the digestion and absorption of food polyphenols, especially in the metabolism of high molecular weight polyphenols into bioactive metabolites (24, 25). Once the polyphenols are transported to the colon, they can be hydrolyzed and metabolized by the colonic microflora to produce aromatic acids (26, 27). When polyphenols are degraded in the small intestine or colon, their metabolites enter the liver. From the liver, most of these metabolites are transported to all parts of the body through the portal vein and participate in various metabolic processes involved in methylation, glucuronidation, and sulfation (25). Additionally, some other metabolites are excreted in the form of bile components that are regenerated by intestinal microbial enzymes before reabsorption, and other unabsorbed metabolites are excreted in the feces (28).

## Molecular Mechanisms of Polyphenols in Colon Cancer

**Figure 2:**
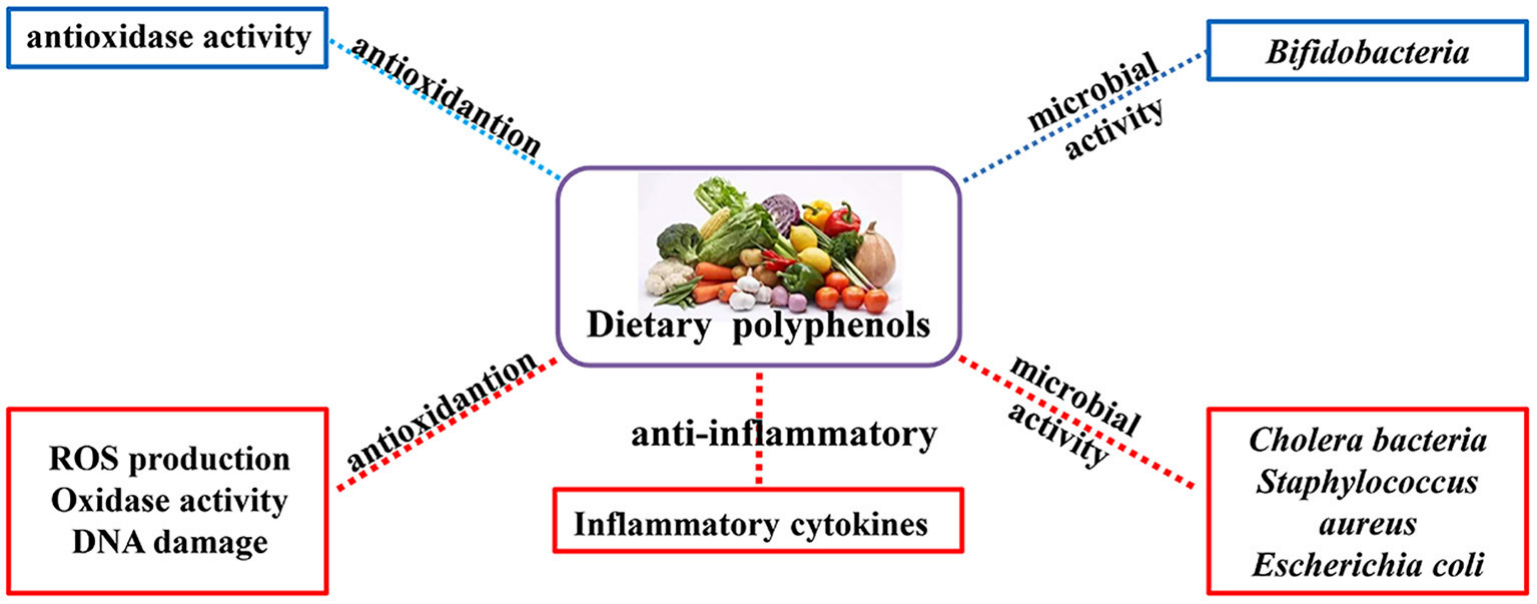
Physiological and nutritional functions of natural polyphenols. The biological functions of polyphenols are mainly antioxidant, anti-inflammatory and regulation of intestinal microorganisms (Red indicates inhibition; blue indicates promotion).

**Figure 3:**
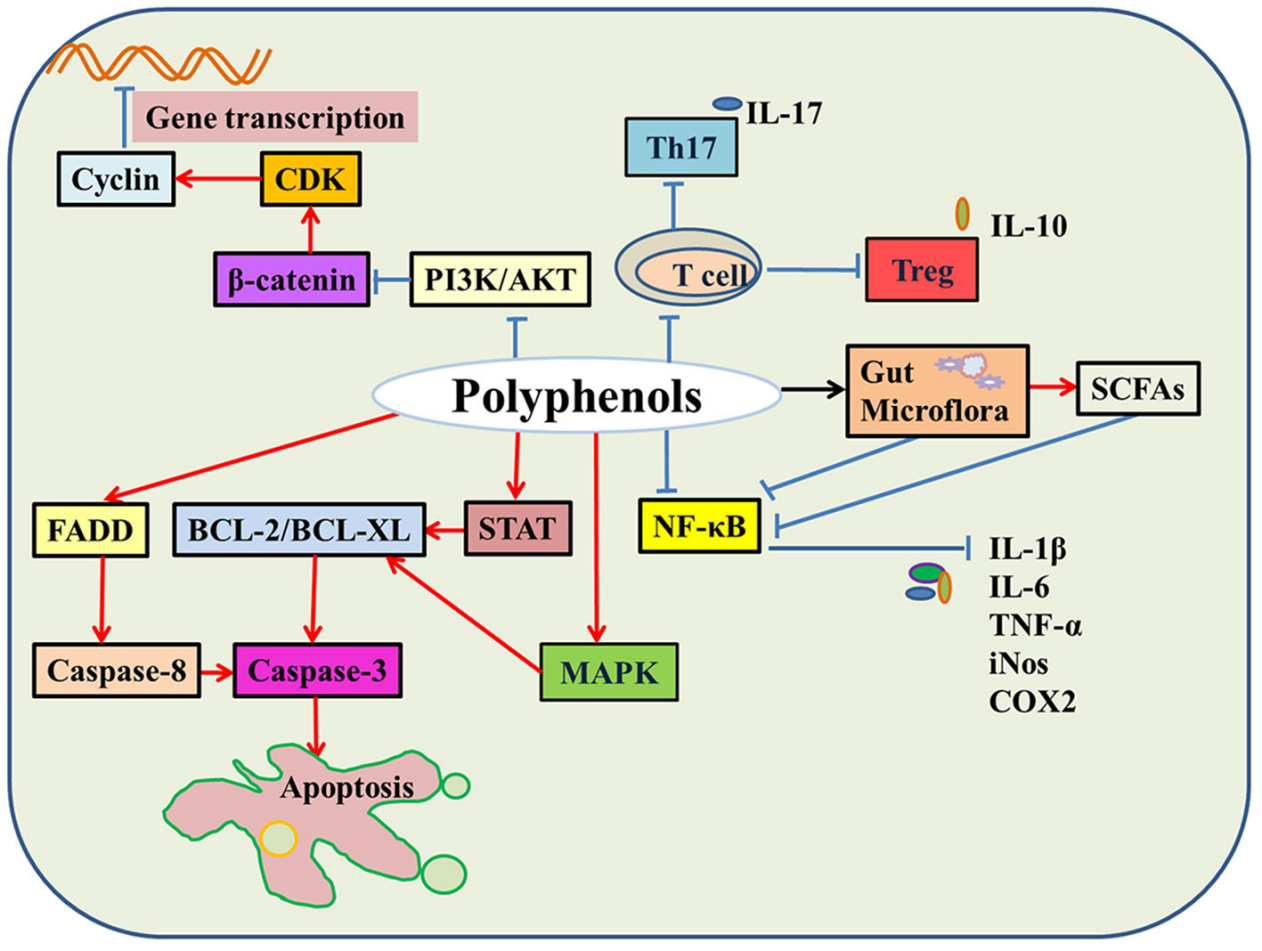
The molecular mechanisms of natural polyphenols in colon cancer. In colon cancer cells, natural polyphenols upregulate the expression of BCL and FADD protein and promote cell apoptosis. Natural polyphenols also downregulate PI3K/AKT and β-catenin signaling pathways and then hinder cell division. Moreover, natural polyphenols inhibit the activity of immune cells, regulate gut microbes leading to down-regulation of the NF-κB signaling pathway, thereby reducing the release of inflammatory factors, such as IL-6, IL-1β, and TNF-α (Red indicates promotion; blue indicates inhibition; black indicates regulation).

It is well-established that natural polyphenols have been reported as an important regulator of intestinal health, including maintaining intestinal nutrient transport and absorption, improving microorganism homeostasis, and promoting growth and development in animals and humans (Figure 2). Recently, an increasing number of studies have shown the regulatory mechanism of polyphenols in the prevention and treatment of colon cancer (29), the details of which are shown in Figure 3 and Table 2.

**Table 2:** The study of a variety of polyphenols from different sources in colon cancer.

**Polyphenols**	**Model**	**Anticancer mechanism**
Pterostilbene (30)	Colon carcinogenesis model in rats	Aberrant crypt foci formation, TNF-α, IL-1β, and IL-4↓ iNOS, COX-2, pGSK-3β, Wnt/β-catenin, VEGF, cyclin D1, and MMPs↓; Ras, PI3K, and EGFR↑
Pterostilbene (31)	HT-29	β-catenin, cyclin D1, and c-MYC↓
Cocoa polyphenols (32, 33)	Caco-2	Cyclooxygenase (COX)-1↑ NF-κB, iNOS, TNF-α, and pJNK↓
Red wine polyphenols (34)	SNU-C4	Bax, Caspase-3↑ Bcl-2↓
Resveratrol (35, 36)	HCT116	Caspase-3↑, PCNA, AKT1/2, pAKT1/2, PI3K/AKT, and Wnt/β-catenin↓
Tea polyphenols (37)	SW480, HT-29	JAG1, MAFA, HES1, MT2A, BAX, and p38↓
Olive oil polyphenols (38)	Caco-2	P38, JNK1/2, NF-κB↑, iNOS↓
EGCG (39, 40)	HCT15, HT-29, CaCo-2	Caspase 9, caspase-3↑ ERK, pERK, EGF, MMP-7, and MMP-9↓
Quercetin (41)	HT-29, HCT-116, SW480	Wnt-β-catenin, Cdc-2, and p21↑ cyclin A↓

### Polyphenols as Efficient Regulators of Oxidative Stress

Owing to the presence of active phenolic hydroxyl groups, polyphenols are considered to be efficient hydrogen donors, reducing agents, and metal chelators, which play an important role in antioxidant function. Some studies have shown that polyphenols can form Fe^3+^ with Fe^2+^ because of the chelating potential of hydroxyl groups, and then inhibit the damage of Fe^2+^ to DNA (42). Other studies have demonstrated that polyphenols can inhibit the production of nicotinamide adenine dinucleotide phosphate hydrogen (NADPH) oxidase (NOX), thereby decreasing the production of ${\text{O}}_{2}^{-}$ in vascular endothelial cells (43). Additionally, polyphenols can reduce the synthesis of mitochondrial ATP by blocking the mitochondrial respiratory chain and ATPase. The main reason for these effects of polyphenols may be that phenolic hydroxyl accepts electrons to form a more stable phenoxy radical, which can protect cellular components from oxidative damage and reduce the risk of various diseases (44, 45). García-Lafuente et al. found that flavonoids could alleviate the excess production of free radicals by inhibiting the activity of pro-oxidant enzymes and reactive oxygen species (ROS) production (29). Studies have reported that gallic acid eliminates excess ROS by stimulating the activity of antioxidant enzymes (e.g., glutathione and superoxide dismutase), inhibiting pro-oxidant enzymes (xanthine oxidase, cyclooxygenase) and protecting lipid peroxidation (42, 46). Erlank et al. verified that resveratrol or curcumin activated the Nrf2/ARE signaling pathway to alleviate oxidative stress by regulating the expression of key genes associated with antioxidant enzymes and electrophilic reaction elements (35). Moreover, Chedea et al. found that dietary grape pomace polyphenols supplementation increased duodenal superoxide dismutase (SOD) activity and colonic CAT and GSH-PX activity in weaned piglets, while it decreased lipid peroxidation in both duodenum and colon to improve the antioxidant ability (47). Kudingcha polyphenols could have great effect on improving the antioxidant capacity of mice with colitis, the mechanism is that Kudingcha polyphenols treatment could significantly decrease MDA level and increase glutathione (GSH) activity, as well as increased the expression of antioxidant related genes including CuZnSOD, MnSOD, GPX1/4, and CAT in colon (48). However, many reports also revealed that colon cancer cell was easily susceptible to oxidative stress and then resulted in cell apoptosis and growth inhibition (49). For example, polyphenols (e.g., epigallocatechin gallate and curcumin) administration would lead to the excessive production of reactive nitrogen species (ROS/RNS), which could damage the cell structure and function (50, 51).

### Polyphenols as Efficient Regulators of Cell Proliferation and Apoptosis

The characteristics of colon cancer cells are unlimited proliferation, dedifferentiation and evasive apoptosis. Zamora-Ros et al. reported that phenolic acid intake was negatively correlated with colon cancer risk in men, suggesting that dietary phenolic acid could lower the risk of colon cancer (52). For example, some studies have reported that natural polyphenols could reduce the number, weight, and volume of tumors and modify the activity of histone acetyltransferase, histone deacetylase, and methyltransferase; thereby upregulating the expression of tumor suppressor genes in cancer cells (9, 53, 54). Metabolic changes are one of the most important markers of cell carcinogenesis, which is consistent with a study that reported a new type of polyphenols having inhibitory effects on glycolysis, mitochondrial respiration and ATP production in colon cancer, while activating 5′ AMP-activated protein kinase (AMPK) and inducing cell death (55). Moreover, with tannic acid supplementation, glycolysis was reduced by inhibiting pyruvate kinase activity, resulting in colorectal cancer (CRC) cells proliferation repression (56). Resveratrol increases ROS production via mitochondrial electron transport chain overload, which ultimately affects cell apoptosis or necrosis and even increases cell death (57, 58).

In the HCT116 cell inflammatory model, resveratrol treatment upregulated the expression of caspase-3 and tumor suppressor factor (PTEN), downregulated the expression of proliferating cell nuclear antigen (PCNA), AKT1/2, pAKT1/2, and pGSK3β, and modulated PI3K/AKT and Wnt/β-catenin signaling pathways to regulate cell proliferation and apoptosis (59). The Wnt/β-catenin signaling was abnormally overexpressed in colorectal cancer and SW480 colon cancer cells; however, tea polyphenols administration effectively inhibited the Wnt/β-catenin signaling pathway to reduce the proliferation of colon cancer cells by activating the GSK3β gene (60). Zeng et al. confirmed that the addition of resveratrol upregulated the expression of bone morphogenetic protein 7 (BMP7) in colon cancer cells and that the activated BMP7 inhibited the PI3K/AKT signaling pathway, which induced the apoptosis of colon cancer cells and exerted anticancer effects (61).

Some studies also found that the effect of resveratrol on the tumor cell cycle was mainly in the occurrence of G2 and S phase arrest. The G2 phase arrest was achieved by inhibiting cyclin-dependent kinase-7 (CDK7) kinase and cyclin-dependent kinase p34 (CDC2) kinase, while the S phase arrest was achieved by inhibiting the activity of CDK2 and CDK4 and DNA synthesis (62). Delmas et al. demonstrated that resveratrol depended on the conformational changes of the Bax and Bak proteins to control cell apoptosis, which also led to the redistribution of the transmembrane Fas receptor in plasma membrane rafts (63). Some reports have also demonstrated that flavonoids activate the mitogen activated protein kinase (MAPK) cascade cell signaling pathway to restore the redox state of colon cancer cells and reduce the expression of Bax to prevent oxidative stress-induced mitochondrial transition pore complex opening (64, 65).

Benhalilou et al. revealed that *Origanum marjorana* ethanolic extract exerted a cytotoxic effect on colon cancer cells by inducing mitotic arrest, activating autophagy and regulating the caspase 3 and 7-dependent extrinsic apoptotic pathway. It has been further evaluated that the main components of the *O. marjorana* ethanolic extract are phenols by the high performance liquid chromatography–mass spectrometry (HPLC–MS) analysis (66). Saadatdoust et al. found that cocoa induced cell apoptosis by activating caspase-3 and inhibiting the activation of STAT3 signaling pathways in a mouse model (67). Hu et al. reported that PPEP, a polyphenol-rich extract isolated from edible mushrooms, effectively downregulated the expression of cell cycle-related signaling proteins (e.g., cyclin B and cyclin E) and upregulated the expression of cell apoptosis-related signaling proteins (e.g., caspase-3) in human colon cancer cells, which further caused considerable cell cycle arrest and cell apoptosis (68). These results indicate that natural plant polyphenols could affect glycolysis, mitochondrial respiration, and other metabolic processes, resulting in blocked DNA synthesis, cell cycle arrest, and cell apoptosis.

### Polyphenols for the Maintenance of the Gut Microflora

Gut microflora refers to the large number of microflorae that exists in the intestinal tract of animals, including probiotics, harmful bacteria, and neutral bacteria (69) to maintain intestinal health. In general, probiotics, like *Bifidobacteria* and *Lactobacillus* can activate the host immune system to resist a variety of intestinal diseases, such as IBD, irritable bowel syndrome (IBS) and colon cancer (70). It is evident that natural polyphenols could significantly upregulate the abundance of *Bifidobacteria* and *Lactobacillus* but inhibit the abundance of the pathogenic bacteria *Clostridium* and *Escherichia coli* in order to defend the host against colitis (71–73). It is possible that the use of plant polyphenols can cause the morphological damage to intestinal pathogenic bacteria and disrupt microbial metabolism while protecting the probiotics from damage (74–77). Notably, many studies have reported that there is a crosstalk between polyphenols and their metabolites and intestinal microorganisms (78). Short-chain fatty acids (SCFAs) are produced by the intestinal flora, which serve as the main energy substrate and play an important role in the regulation of intestinal health (79). A recent report has shown that citrus flavanones could also inhibit the growth of pathogens, increase the production of symbiotic bacteria (such as *Bifidobacterium* and *Lactobacillus* species), and stimulate the production of SCFAs to alleviate intestinal inflammation (80). Janssen and Kersten (2015) have demonstrated that natural polyphenols participate in the SCFA metabolism of intestinal microbes to affect energy absorption in the host (81).

An imbalance of microbes in the gut increases the risk of obesity and colon cancer (82). And obesity will cause mitochondrial dysfunction and reduce oxygen consumption rate in colon cells, which in turn increases susceptibility to colon cancer (83). Dysregulation of intestinal flora activates inflammatory pathways (e.g., NF-κB, TLR, and STAT3) and causes the release of bacterial toxins to accelerate the development of colon cancer. However, many studies have reported that administration of polyphenols could improve the composition of colonic microorganisms or the concentration of metabolites as well as downregulate NF-κB and TLR signaling pathways to inhibit the development of colon cancer (84, 85). Stevens et al. showed that citrus flavanones influenced the abundance of *Bifidobacterium, Lactobacillus*, and *Staphylococcus aureus* to alleviate intestinal inflammation (80). Moreover, polyphenols stimulated the production of SCFAs in colon cancer (86), and SCFAs could help shape the stable intestinal microbiological environment. Furthermore, natural polyphenols control the production of lactic acid in gut microbes by regulating the expression of SCFA transporters (86, 87). Quercetin and metabolites of chlorogenic acid produced by bacteria, 3, 4-dihydroxyphenylacetic acid, and 3-(3,4-dihydroxybenzene)-propionic acid can reduce the expression of cyclooxygenase-2 in colorectal adenoma cells and regulate the DNA damage induced by cumene hydroperoxide (88). In other words, polyphenolic metabolites produced by the intestinal microflora may directly promote the anticancer action of polyphenols.

### Polyphenols as Efficient Suppressors of Inflammation

Polyphenols regulate the infiltration of immune cells into tumor tissues to enhance immune responses and counteract immune escape, thereby alleviating colon cancer (82). In a CRC experimental model, natural polyphenol supplementation modulated the tumor-associated immunosuppressive microenvironment and inhibited the growth of CRC cells by reducing the colonic infiltration of CD3^+^ T cells and the number of CD25-FoxP3 regulatory T cells, increasing the mucosal CD4^+^ T and B cells (89–92). Besides, in a multiple intestinal neoplasia/+ (ApcMin) mice model, cloudberry administration reduced the proportion of CD3^+^ T lymphocytes, FoxP3^+^ regulatory T lymphocytes, and CD45R^+^ B lymphocytes, thereby attenuating intestinal inflammation (93). A previous study demonstrated that curcumin supplementation inhibited the suppressive activity of Treg cells by downregulating the production of TGF-β and IL-10, which could enhance the ability of effector T cells to kill cancer cells (94). The increase in Th17 cells in patients with metastatic colorectal cancer is associated with poor prognosis (95). The Th17 cells enhanced tumor cell resistance to apoptosis and proliferation by promoting Il-17 secretion, and the Th17 cells were reduced after red wine extract treatment (red wine polyphenols), which might have been the cause of delayed tumor growth (96, 97). Furthermore, treatment with resveratrol could regulate the balance of Treg/Th17 and the levels of intestinal mucosal cytokines, including IL-10, TGF-β, IL-6, and IL-17, in an ulcerative colitis mouse model (98). Cocoa has been demonstrated to affect the gut immune responses by increasing the percentage of γδ T cells and weakening the effect of IgA in young rats (99). Oz et al. found that green tea polyphenols could decrease the levels of inflammatory cytokines TNF-α and IL-6 and improve antioxidant enzyme levels in patients with colitis (100). Emerging evidence indicates that the rat with colitis has the properties for the increased intestinal permeability and impaired barrier function; flavonoids treatment could maintain intestinal permeability and increase the expression and tight junction protein to alleviate colitis (80, 101).

Recent studies have demonstrated that polyphenols exert therapeutic and preventive effects against colon cancer by modulating multiple signaling pathways both *in vitro* and *in vivo*, such as the NF-κB pathway, MAPK pathway, PI3K/AKT pathway, Wnt/β-catenin pathway, and c-Jun N-terminal kinase (JNK) pathway (102, 103). The NF-κB pathway, a major inflammatory signaling pathway, promotes the production of iNOS, COX-2, and proinflammatory cytokines (IL-1, IL-2, IL-6, and TNF-α) to stimulate the inflammatory response (104). Many reports have shown that COX-2 is expressed in large quantities in colon tumor cells, and COX-2 silencing or knockout inhibits the invasive potential of CRC cells and decreases the metastatic potential of colorectal tumor cells (105, 106). Previous studies have shown that curcumin reduced the expression of COX-2, iNOS, and TNF genes and inhibited the protein expression of NF-κB, cJNK, protein tyrosine kinases and protein serine/threonine kinases (GSK-3β), further depressing tumorigenesis (107). In addition, cocoa polyphenols decreased the expression of the inflammatory marker COX-2 and nitric oxide (NO) synthase pro-inflammatory enzymes and suppressed NF-κB nuclear translocation and JNK phosphorylation to relieve colon cancer (32, 108). In contrast, gallic acid equivalent (GAE) treatment increased intracellular ROS production in human cancer colon fibroblast cells and decreased the expression levels of TNF-α, IL-1β, IL-6, and NF-κB to suppress cell proliferation (109). However, in human non-cancer colon fibroblast cells, GAE promoted anti-inflammatory activities, which was accompanied by the reduction of intracellular ROS and the inhibited expression of TNF-α, IL-1β, IL-6, and NF-κB (109). These results suggest that polyphenols may play a key role in preventing colon cancer by modulating the production of inflammatory cytokines and the activation of immune cells and key signaling pathways to regulate the inflammatory tumor microenvironment.

## Application of Polyphenols in Disease Prevention

Polyphenols have a wide range of applications in the food, medical, and pharmaceutical fields owing to their multiple health benefits, including antioxidant and antibacterial activities, cancer prevention, antiradiation effects, and immune enhancement. It is known that colon cancer cells develop drug resistance under chemotherapy conditions, but polyphenols intervention, like curcumin, which can promote apoptosis of cancer stem cells and reduce drug resistance (41). However, polyphenols have significant anticancer potential in *in vitro* cell culture and *in vivo* animal models, there were only very few clinical trials to prove the effectiveness of dietary polyphenols in the prevention and treatment of cancer due to challenges, such as ineffective stability, and low bioavailability. The utilization of polyphenols is low due to the metabolism of host enzymes (e.g., lactase-phlorizin hydrolase and cytosolic β-glucosidase) and intestinal microorganisms (110). Therefore, we should focus on improving the bioavailability of polyphenols in future studies. For example, Tabrez et al. showed that nanoencapsulation can extend circulation, improve localization, enhance efficacy, and reduce the chance of multidrug resistance (111). Thus, polyphenol nanomaterials are promising in the clinical treatment of colon cancer.

Besides, some studies have shown that natural polyphenols have effects on various bacteria, fungi, and yeasts, such as the cholera *bacteria, S. aureus, E. coli*, and other common pathogenic bacteria (25). Flavonols are commonly used to treat *Microsporum gypseum, Trichophyton mentagrophytes*, and *Trichophyton rubrum* to alleviate the allergic reaction of bacterial and fungal dermatitis (112). Proanthocyanidins can prevent viruses from entering host cells, such as influenza A virus and herpes simplex virus type 1 (113). Different kinds of polyphenols (i.e., flavanones, green tea polyphenols, red wine polyphenols, and resveratrol) have been valued primarily as crucial regulators in both *in vivo* and *in vitro* inflammatory models and can be used as potential agents to treat diseases, such as diabetes, obesity, and cardiovascular diseases (27). For example, flavanones, an effective inhibitor of low-density lipoprotein, might provide a protective effect against cardiovascular and chronic inflammatory diseases (114).

## Conclusions and Perspectives

Numerous studies have reported that natural plant polyphenols are critical modulators of intestinal flora, inflammation, cell cycle, and apoptosis in colon cancer, making them a potential therapeutic agent. To date, an increasing number of dietary plant polyphenols has been used as anticancer agents to prevent and treat colon cancer or other intestinal diseases. Furthermore, the regulatory mechanisms of natural plant polyphenols on intestinal flora, inflammation, and cancer-cell proliferation and death have become clear in tumor cells, including the STAT, NF-κB, Wnt/β-catenin, PI3K/Akt, and apoptosis-related signaling pathways.

Natural polyphenols are promising candidates for the prevention and treatment of colon cancer. However, many biochemical mechanisms and clinical applications of plant polyphenols in colon cancer remain to be further resolved, such as metabolism in the gastrointestinal tract and visceral tissue, synergistic effect of polyphenols with drugs or diverse chemotherapeutics, and targeted therapies. Importantly, for the use of polyphenols as a potential targeted therapy, the targeted regulatory sites should be determined and targeted inhibitors developed in the future. Therefore, the relationship between colon cancer and plant polyphenols needs to be further clarified. In addition, large-scale biological production of dietary plant polyphenols has not been extensively investigated and will be one of our future research projects.

## Author Contributions

JLo and PG wrote the manuscript. TL, LH, JLo, XHu, ZD, FL, and LY discussed the topic. JLi, QL, XHe, and LH revised the manuscript. All authors contributed to the article and approved the submitted version.

## Conflict of Interest

XHe was employed by the company Changsha Green Leaf Biotechnology Co., Ltd. The remaining authors declare that the research was conducted in the absence of any commercial or financial relationships that could be construed as a potential conflict of interest.

## Abbreviations

IBD: inflammatory bowel disease
SOD: superoxide dismutase
NADPH: nicotinamide adenine dinucleotide phosphate hydrogen
ROS: reactive oxygen species
AMPK: 5′ AMP-activated protein kinase
CRC: colorectal cancer
PTEN: tumor suppressor factor
PCNA: proliferating cell nuclear antigen
BMP7: bone morphogenetic protein
CDK7: cyclin-dependent kinase-7
HPLC–MS: high performance liquid chromatography–mass spectrometry
SCFAs: Short-chain fatty acids
MAPK: activated protein kinase
JNK: c-Jun N-terminal kinase
GSK-3β: serine/threonine kinases
NO: nitric oxide
GAE: gallic acid equivalent
PPARγ: peroxisome proliferator-activated receptor gamma.
